# Linking Work-Life Occupational Exposures With Distress and Mortality Before and After Retirement

**DOI:** 10.1093/geroni/igaa057.1425

**Published:** 2020-12-16

**Authors:** Sarah Laditka, James Laditka, Ahmed Arif

**Affiliations:** University of North Carolina at Charlotte, Charlotte, North Carolina, United States

## Abstract

Mental health problems have surpassed musculoskeletal injuries as causes of work disability. Workers in certain occupations may have high risks for mental health problems and premature death even after retirement. People in high risk occupations for many years may be particularly vulnerable, along with groups with higher health risks such as rural residents. Little research examines their occupation-related risks. No research has examined how occupational exposures affect mental health in retirement. We studied these life course risks using the nationally representative Panel Study of Income Dynamics, following participants 36 years beginning 1981, with annual measures of occupation and distress (n=16,994; 129,880 occupation measures; 415 deaths). We estimated hazards of developing distress in occupations hypothesized to have high and low distress risks, adjusted for factors associated with occupational choice and mental health including age, education, income, race/ethnicity, sex, childhood and midlife health, and family trauma. Compared to low risk occupations, working in high risk occupations was associated with 20% elevated odds of distress (adjusted odds ratio, OR 1.20, 95% confidence interval, CI 1.13 1.28) and 55% elevated risk of death (hazard ratio 1.55, CI 1.11-2.16). Each additional year in a high risk occupation increased the odds by 5%. Rural residents had the highest occupation-related distress risk (adjusted OR 3.05, CI 2.39-3.97). At ages 70+ each additional past exposure year was associated with 2% higher distress risk (p<0.05), and 4.6% higher mortality (p<0.05). Workers in certain occupations have high risks of psychological distress and death, even after retirement.

